# A 25-years dataset of ageostrophic, Ekman and Stokes surface currents in the Mediterranean Sea (AGESC-Med)

**DOI:** 10.1016/j.dib.2023.109804

**Published:** 2023-11-11

**Authors:** Verónica Morales-Márquez, Ismael Hernández-Carrasco, Alejandro Orfila

**Affiliations:** a Université de Toulon, Aix Marseille Univ., CNRS, IRD, MIO, Toulon, France; b Mediterranean Institute for Advanced Studies (CSIC-UIB), 07190 Esporles, Balearic Islands, Spain

**Keywords:** Ocean surface velocity, Ekman velocity, Stokes velocity, Mediterranean Sea

## Abstract

A 25-years surface velocity data-set for the Mediterranean Sea is presented in this paper. The velocity data is obtained using a modified Ekman model which allows us to obtain an analytical solution for the surface currents using satellite altimetry and available wind and waves data from synoptic observations. The new database consists of 6-hourly ocean surface velocities (integrated over one-meter depth) including the geostrophic component and the Ekman and Stokes velocity components driven by the wind and waves, between 1993 to 2018, and covering the whole Mediterranean Sea with a spatial resolution of $1/8^{\circ }$. The resulting Ageostrophic, Ekman and Stokes Currents in the Mediterranean basin (AGESC-Med) was validated validated with real drifters collected by the Italian National Institute of Oceanography and Experimental Geophysics (OGS). The AGECS-Med product improves the currently available sea surfave velocity fields obtained from altimetry, as well as analyze the meoscale ageostrophic dynamics induced by wind and waves.

Specifications Table

**Table:** 

Subject	Oceanography
Specific subject area	Surface currents
Type of data	Raw data files (NetCDF format files)
How the data were acquired	Waves and wind dataset from ERA-Interim Reanalysis and altimeter satellite dataset from CMEMS web platform.
Data format	Raw
Description of data collection	Total, ageostrophic, Ekman and Stokes components of the surface currents velocity fields are computed using a simplified Ekman model considering the steady state of the conservative wave-averaged Boussinesq horizontal momentum equation, within a uniform and steady surface gravity wave field in the presence of surface wind stress and with a small Rossby number.
Data source location	• Absolute geostrophic velocity data are available on the CMEMS web platform [1]https://data.marine.copernicus.eu/products, DOI: 10.48670/moi−00141. • Wind and waves data are available on ERA5 reanalysis [2]https://cds.climate.copernicus.eu/cdsapp#!/dataset/reanalysis-era5-single-levels?tab=form, DOI: 10.24381/cds.adbb2d47
Data accessibility	Repository name: Zenodo Data identification number: https://doi.org/10.5281/zenodo.8289301. Direct URL to data: https://zenodo.org/record/8289301
Related research article	V. Morales-Márquez, I. Hernández-Carrasco, G. Simarro, V. Rossi, A. Orfila, Regionalizing the Impacts of Wind- and Wave-Induced Currents on Surface Ocean Dynamics: A Long-Term Variability Analysis in the Mediterranean Sea, JGR Ocean. 126(9) (2021) e2020JC017104. https://doi.org/10.1029/2020JC017104

## 1 Value of the Data

• This database includes the wind and waves driven ageostrophic component into the total surface velocity in the Mediterranean Sea.
• Oceanographers (physical, civil and marine engineers, biogeochemical and environmental areas), Search and Rescue (SAR) agencies and services, ocean engineers, shipping companies and public administrations can benefit from this data.
• This database can be used to (i) analyze surface dynamics in the Mediterranean Sea; (ii) to study of particles dispersion; (iii) for SAR operations, optimization of shipping routes; (iv) interannual climate analysis; (v) Biogeochemical variability; and (vi) marine ecology studies.

## 2 Objective

This database provides surface velocity dataset in the Mediterranean Sea including geostrophic satellite derived currents, wind and waves giving outputs from an analytical model using observational data. These data is an added value to the current surface velocity field that is based on mesoscale observations. The analysis of the dataset was carried out in a previous publication [3].

The applications of this database are versatile and span various fields of study. Firstly, it can be exploited for in-depth analyses of surface dynamics within the Mediterranean Sea. Researchers and scientists can investigate the intricate patterns and interactions of currents, winds, and waves, gaining a deeper understanding of the region’s oceanography.

Secondly, the dataset proves essential for the study of particle dispersion. Understanding how particles, such as pollutants or marine organisms, move within the water column is crucial for environmental assessments and ecosystem studies. The accurate surface velocity information provided by the database helps in tracing the pathways and behaviors of these particles.

Moreover, the dataset plays a key role in supporting Search and Rescue (SAR) operations. By incorporating precise surface velocity data, SAR missions can be optimized. Similarly, the maritime industry benefits from the database as it facilitates the optimization of shipping routes, taking into account the most current and accurate information on sea currents and winds.

On a larger scale, the database contributes to interannual climate analyses. The availability of consistent surface velocity data over time enables the study of long-term oceanic trends and the impact of climate change on the Mediterranean Sea’s dynamics.

The database also proves to be a valuable resource for exploring biogeochemical variability. Oceanic currents play a significant role in the distribution of nutrients and chemicals, affecting marine life and ecosystems. Researchers can utilize the dataset to delve into these intricate relationships, ultimately contributing to a comprehensive understanding of the Mediterranean Sea’s biogeochemical processes.

Finally, marine ecology studies benefit immensely from the provided data. The movement of water masses influences the distribution of marine species, their habitats, and migration patterns. The surface velocity dataset supports ecological investigations by offering insights into how ocean dynamics shape and influence the biodiversity of the Mediterranean Sea.

This database of surface velocity data for the Mediterranean Sea serves as a cornerstone for diverse scientific endeavors. Its multidisciplinary applications encompass oceanography, environmental assessment, maritime operations, climate research, biogeochemical studies, and marine ecology, making it an invaluable resource for advancing knowledge and understanding of this vital marine region.

## 3 Data Description

The presented dataset contains 26 files where we can find the zonal and meridional components of the total ($U_{T}$), ageostrophic ($U_{a}$), Ekman ($U_{E}$) and Stokes ($U_{S}$) velocity components integrated over the upper one meter depth [3,4], as well as the longitude, the latitude, depth and time for each specific month. Each NetCDF file includes all the variables for 1 year. The naming conventions are:

• time: time in days.
• longitude: longitude in degrees East.
• latitude: latitude in degrees North.
• ut: Total zonal velocity in m/s.
• vt: Total meridional velocity in m/s.
• ua: Ageostrophic zonal velocity in m/s.
• va: Ageostrophic meridional velocity in m/s.
• ue: Ekman zonal velocity in m/s.
• ve: Ekman meridional velocity in m/s.
• us: Stokes zonal velocity in m/s.
• vs: Stokes meridional velocity in m/s.

The variables have a time resolution of 6 h (data is provided each day at 00 h, 06 h, 12 h and 18 h) from January, $1{}^{st}$ 1993 at 00 h to June, 10th 2018 at 18 h with a spatial resolution of $0.125^{\circ }$ in a mesh of 344×128 nodes covering the Mediterranean Sea. Bottom left corner coordinates are $-6^{\circ }$E; $30.125^{\circ }$N and top right corner coordinates are $36.875^{\circ }$E; $46^{\circ }$N.

## 4 Experimental Design, Materials and Methods

This section introduces a new surface velocity dataset derived after solving an analytical modified Ekman model. The model takes into account the impact of surface wind, wave stress, and the Coriolis force [5,6].

Before presenting the model equation, let’s outline the simplifications adopted. We start by applying the conservative wave-averaged horizontal Boussinesq approximation to account for the influence of a steady and uniform surface gravity wave field [7]. Additionally, we assume that the system is affected by Coriolis forces, allowing us to work with a small Rossby number. These simplifications lead to a simplified Ekman model, which excludes non-linear advection terms and takes into consideration the absence of small-scale Langmuir vortexes.

The momentum equation is expressed in complex notation, where U≡u+iv and $\nabla =\frac{\partial }{\partial x}+i\frac{\partial }{\partial y}$:

(1) $$if\;U_{T}=-\frac{1}{\rho_{w}}\nabla P+\frac{1}{\rho_{w}}\frac{\partial \tau }{\partial z}-if\;U_{s}-T_{wds}.$$

In this equation, $\rho_{w}$ represents water density, $T_{\mathrm{wds}}$ indicates the momentum transfer from waves to mean flow due to dissipation of wave energy, and $U_{T}$ signifies the total velocity flow. The wave-induced Stokes drift is denoted as $U_{s}$ defined as $U_{s}=a^{2}\omega ke^{2kz}\hat{k}$ in [8], where k is the wave-number magnitude with $\hat{k}$ as its unity vector, a represents the wave amplitude and ω is the wave-frequency number. Assuming a deep water monochromatic wave, the relation between k and ω is constrained by $k=\omega^{2}/g$. The Stokes-Coriolis force is represented by $\mathrm{if}\;U_{s}$, while vertical mixing results from wind and wave stress τ, and horizontal mixing is neglected.

The total velocity $U_{T}$ is approximated as the sum of geostrophic velocity $U_{g}$ (represented as $\left[ifU_{g}=-\frac{1}{\rho_{w}}\nabla P\right]$ in Eq.  (1)) and wind- and wave- induced ageostrophic velocity $U_{a}$. We obtain the geostrophic velocity using altimetry data. By this way, small-scale contributions to the momentum equation are averaged out, rendering the inclusion of the Stokes drift contribution $U_{s}$ in the pressure gradient term unnecessary. Hence, the effect of waves on ocean currents can be modeled through a modification of the surface boundary condition [6], [9], where the ageostrophic velocity field includes the Stokes-Coriolis, wave radiation and mixing effects, taking into account that Ekman depth is much larger than the Stokes layer depth, as it is the case of the Mediterranean sea [10]. Consequently, the equation for the ageostrophic current can be approximated as,

(2) $$\begin{array}{c} if\;U_{a}=\frac{\partial }{\partial z}\left(A_{z}\frac{\partial U_{a}}{\partial z}\right)-if\;U_{s}-T_{\mathrm{wds}}, \end{array}$$

where the Ekman-Stokes stress is $\tau =\rho A_{z}\left(z\right)\frac{\partial U_{a}}{\partial z}$, being $A_{z}$ the vertical viscosity profile, considered uniform in the whole basin [3], [5], [6] and calculated as $1.2\cdot 10^{-4}U_{10}^{2}$ $m^{2}s^{-1}$ [11], [12]. The momentum transfer from waves to the mean flow due to dissipation of wave energy ($T_{wds}$) is neglected.

We solve the ageostrophic momentum equation (Eq. (2)) as a two-point boundary value problem where we have the modified Ekman-Stokes stress and vanishing conditions at z=0 and at z=−∞, respectively. In this way, the analytical solution is:

(3) $$\begin{array}{c} U_{a}\;\left(z\right)\;=\frac{\tau_{w}}{\rho_{w}A_{z}m}e^{\mathrm{mz}}+\frac{\frac{\partial S}{\partial X}}{\rho_{w}A_{z}m}e^{\mathrm{mz}}+\frac{m^{2}U_{s0}}{4k^{2}-m^{2}}e^{2\mathrm{kz}}-\frac{2\mathrm{km}U_{s0}}{4k^{2}-m^{2}}e^{\mathrm{mz}}, \end{array}$$

where $\tau_{w}$ is the wind stress, computed as $\tau_{w}=\rho_{a}C_{D}u_{10}u_{10}$, being $\rho_{a}=1.2\text{kg}/m^{3}$ the air density; $u_{10}$ the 10-m wind speed and $C_{D}$, the neutral drag coefficient specified in [13] as, $C_{D}=\left(2.7/u_{10}+0.142+0.0764u_{10}\right)/1000$; $m=\sqrt{if/A_{z}}=\;(1+i)\;\sqrt{f/\;(2A_{z})\;}$; $\frac{\partial S}{\partial X}$ the radiation stress due to the waves at the sea surface and $U_{s0}=U_{s(z=0)}$.

Different components of ageostrophic velocity (3) can be distinguished as follows [6]:

• Classical Ekman component: $U_{E}\;(z)\;=\frac{\tau_{w}}{\rho_{w}A_{z}m}e^{mz}$,
• Surface current induced by wave radiation stress: $U_{\tau_{s}}\;\left(z\right)\;=\frac{\frac{\partial S}{\partial X}}{\rho_{w}A_{z}m}e^{\mathrm{mz}}$,
• Stokes component: $U_{S}\;(z)\;=\frac{m^{2}U_{s0}}{4k^{2}-m^{2}}e^{2kz}$,
• Ekman-Stokes component: $U_{ES}\;(z)\;=-\frac{2kmU_{s0}}{4k^{2}-m^{2}}e^{mz}$.

We conducted an integration of $U_{a}$ over the upper 1 m, taking into account that the influence of the Stokes component extends to the Stokes layer depth ($d_{S}=1/\left(2k\right)$), which is smaller than the Ekman layer depth ($d_{E}=1/m$) where the remaining ageostrophic components operate. Given the typical mean depth of the Stokes layer in the Mediterranean Sea is less than 2 meters, our approach ensures the preservation of all ageostrophic components within the uppermost meter of the ocean surface.

**Fig. 1:**
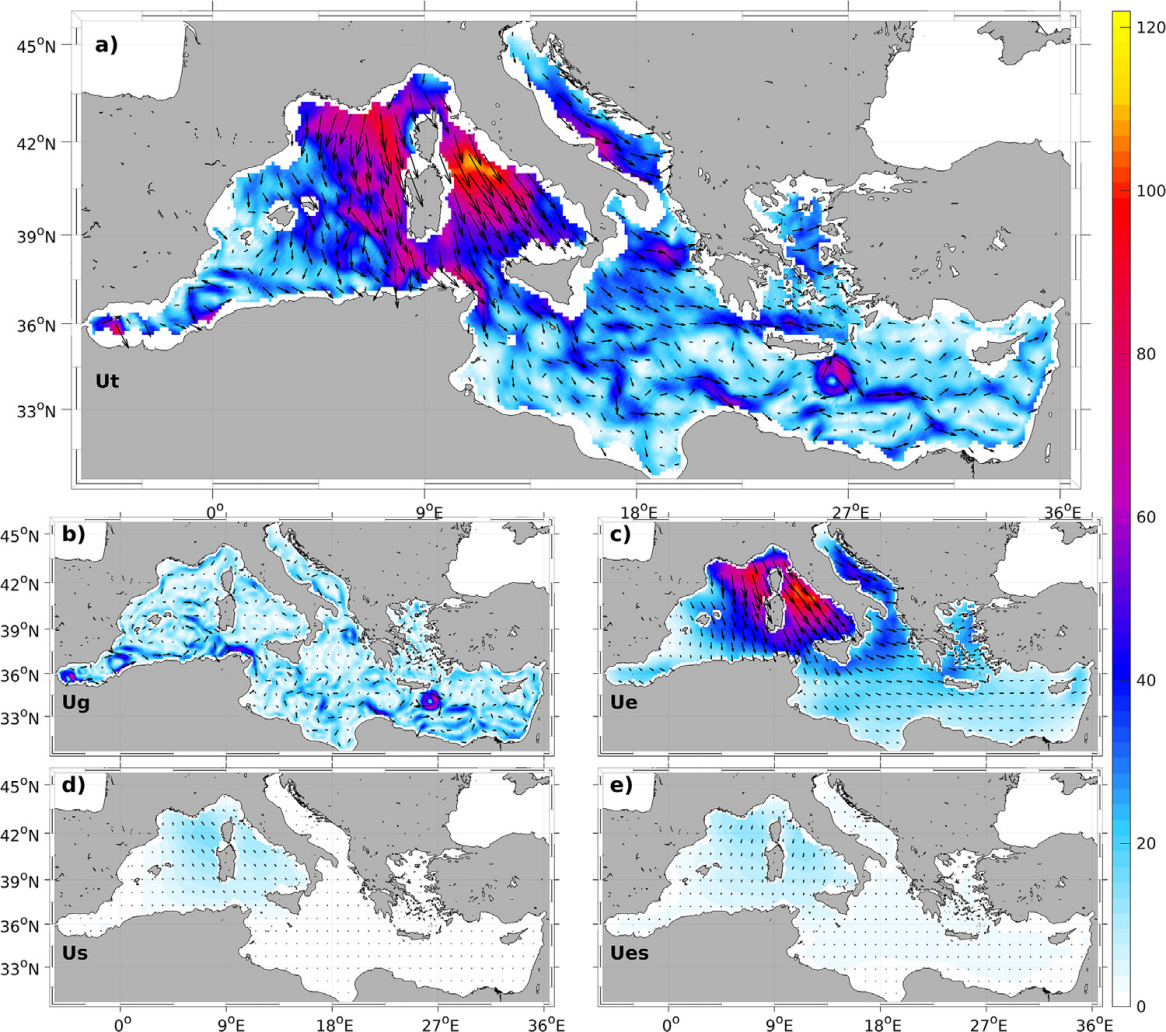
a) Total, b) Geostrophic, c) Ekman, d) Stokes and e) Ekman-Stokes velocity fields in cm/s for the 28th of December of 1999 at 12 UTC. The black arrows represent the direction of the velocity fields.

The primary datasets for waves and 10,-m height wind are extracted from the ERA-Interim reanalysis product (integrated inside ERA5 from June 2023, https://cds.climate.copernicus.eu/cdsapp#!/dataset/reanalysis-era5-single-levels?tab=form), using a WAM wave model assimilated with ERS1 satellite wave height data [14]. These products have a temporal resolution of 6 h from 1979 to 2019 and a longitudinal and latitudinal resolution of $1/8^{\circ }$ covering the whole Mediterranean Sea [15]. Geostrophic velocity data are obtained from the Copernicus Marine Environment Monitoring Service (CMEMS) through the product *European Seas Gridded L4 Sea Surface Heights And Derived Variables Reprocessed 1993 Ongoing* (absolute geostrophic velocity fields) [1]. These data have a daily temporal resolution, however we have interpolated it each 6 h, in accordance with the ageostrophic velocities, to compute the total velocity field in a regular mesh of $1/8^{\circ }$ over the entire Mediterranean Sea from January 1993 to June 2018. In Fig. 1 we can see an example of the total surface velocity currents in the Mediterranean Sea for 28th December 1999 at 12 h (top panel) and the velocity for the geostrophy ($U_{g}$), Ekman ($U_{e}$), Stokes ($U_{s}$) and Ekman-Stokes ($U_{\mathrm{es}}$) components.

**Fig. 2:**
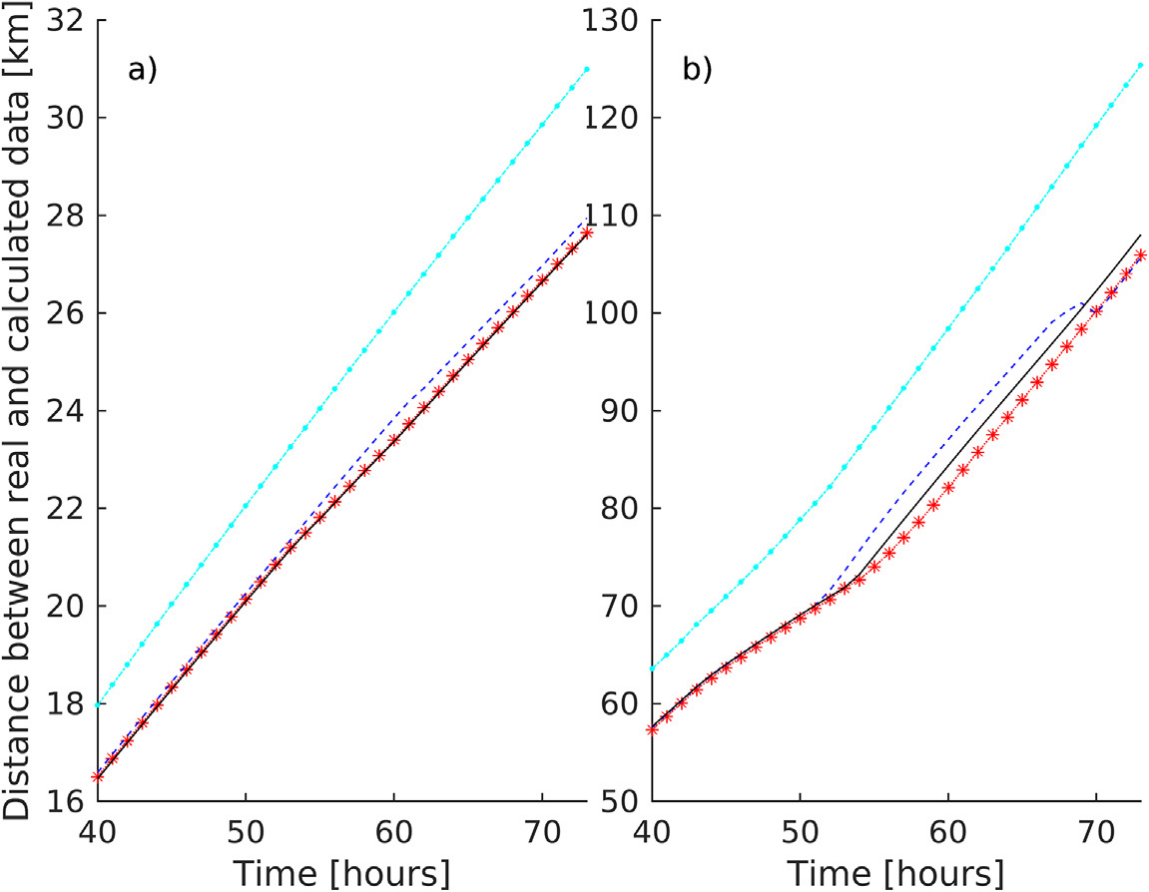
a) Mean and b) maximum distance (in km) between all the available real and virtual drifters’ trajectories as a function of time, D(t), advected in Ug (cian dash-dotted line), $U_{g}\;+\;U_{E}\;+\;U_{\tau_{s}}$ (red star-dotted line) $U_{g}\;+\;U_{E}\;+\;U_{\tau_{s}}\;+\;U_{S}$ (blue dashed line) and $U_{T}$ (black line), 40 h after the initialization in each hourly real drifters location.

The validation of the surface velocity dataset involved 690 surface drifters, comprising 15 from the Surface Velocity Program (SVP) and 675 from the Coastal Ocean Dynamics Experiment (CODE). Deployed between 1994 and 2005 by various Mediterranean Sea institutions, these drifters were collected by the Italian National Institute of Oceanography and Experimental Geophysics (OGS) [16]. Through this validation process, we observed that the average separation between actual and simulated drifter trajectories (calculated using the total, geostrophic, and different ageostrophic components) is minimized when virtual trajectories are determined using the entire velocity field (including currents induced by wind and waves) rather than solely relying on geostrophic velocities (see Fig. 2). Notably, mean and maximum differences between virtual particles advected with the geostrophic velocity field and advected with the total velocity (including also Ekman and Stokes components) and compared to real drifter trajectories are 4 km and 25 km after 72 h respectively This shows the importance of including the high frequency velocity components (driven by the wind and waves).

## Ethics Statements

The study does not involve experiments on humans or animals. All the primary data using in order to develop this model are public data.

## CRediT authorship contribution statement

**Verónica Morales-Márquez:** Methodology, Software, Validation, Formal analysis, Investigation, Resources, Writing – original draft, Visualization, Project administration. **Ismael Hernández-Carrasco:** Conceptualization, Methodology, Validation, Investigation, Resources, Writing – review & editing, Supervision. **Alejandro Orfila:** Conceptualization, Methodology, Validation, Investigation, Writing – review & editing, Supervision, Funding acquisition.

## Data Availability

25-years dataset of Ageostrophic, Ekman and Stokes surface currents in the Mediterranean Sea (AGESC-Med)
